# Moderate-to-deep sedation technique, using propofol and ketamine, allowing synchronised breathing for magnetic resonance high-intensity focused ultrasound (MR-HIFU) treatment for uterine fibroids: a pilot study

**DOI:** 10.1186/s40349-017-0088-9

**Published:** 2017-02-09

**Authors:** Hermanus H. B. Vaessen, F. M. Knuttel, J. M. M. van Breugel, M. E. Ikink, J. M. Dieleman, M. A. A. J. van den Bosch, J. T. A. Knape

**Affiliations:** 1 Division of Anaesthesiology, Intensive Care and Emergency Medicine, University Medical Center Utrecht, Heidelberglaan 100, Huispostnr.: F 04.5.16, 3584 CX Utrecht, The Netherlands; 2 Department of Radiology, University Medical Center Utrecht, Utrecht, The Netherlands

**Keywords:** Deep propofol and esketamine sedation, Safety, MR-HIFU uterine fibroids treatment, Hallucinations and sedation-related complications

## Abstract

**Background:**

Magnetic resonance high-intensity focused ultrasound (MR-HIFU) treatment for uterine fibroids is rapidly gaining popularity as a treatment modality. This procedure is generally uncomfortable, painful, and requires minimal or absence of movement and an MR-HIFU synchronised breathing pattern of the patient. Procedural sedation and analgesia protocols have become the standard practice in interventional radiology departments worldwide. The aim of this study was to explore if a sedation regimen with low-dose propofol and ketamine performed by trained non-medical sedation practitioners could result in relief of discomfort for the patient and in adequate working conditions for MR-HIFU treatment for uterine fibroids.

**Methods:**

In this study, conducted from August 2013 until November 2014, 20 patients were subjected to MR-HIFU treatment of uterine fibroids. Patients were deeply sedated using intravenous propofol and esketamine according to a standardised hospital protocol to allow synchronisation of the breathing pattern to the MR-HIFU. The quality of sedation for MR-HIFU and complications were recorded and analysed. The side effects of the sedation technique, the propofol and esketamine consumption rate, the duration of recovery, and patient satisfaction after 24 h were examined.

**Results:**

A total of 20 female patients (mean age 42.4 [range 32–53] years) were enrolled. Mean propofol/esketamine dose was 1309 mg/39.5 mg (range 692–1970 mg/ 23.6–87.9 mg). Mean procedure time was 269 min (range 140–295 min). Application of the sedation protocol resulted in a regular breathing pattern, which could be synchronised with the MR-HIFU procedures without delay. The required treatment was completed in all cases. There were no major adverse events. Hypoxemia (oxygen desaturation <92%) and hallucinations were not observed.

**Conclusions:**

The use of a specific combination of IV propofol and esketamine for procedural sedation and analgesia reduced the discomfort and pain during MR-guided HIFU treatments of uterine fibroids. The resulting regular breathing pattern allowed for easy synchronisation of the MR-HIFU procedure. Based on our results, esketamine and propofol sedation performed by trained non-medical sedation practitioners is feasible and safe, has a low risk of major adverse events, and has a short recovery time, avoiding a session of general anaesthesia.

**Electronic supplementary material:**

The online version of this article (doi:10.1186/s40349-017-0088-9) contains supplementary material, which is available to authorized users.

## Background

Procedural sedation and analgesia (PSA) has become standard practice to achieve high-quality and efficient conditions for uncomfortable and/or painful intervention radiology procedures worldwide. Over the past decades, the frequency of complex interventional radiology procedures such as magnetic resonance high-intensity focused ultrasound (MR-HIFU) has increased. The use of MR-HIFU to treat tumours was first investigated in 1994 [1]. The development of MR-HIFU ablation is the result of a search for less invasive treatments that can be performed outside the operating room [2]. These treatments are effective and less invasive when compared to surgery, and are associated with less complication in fibroid treatments [3–5]. MR-HIFU has been shown to be an optimal modality for treating uterine fibroids because of their good inherent tissue contrast [6], high absorption of the ultrasound waves, and low blood flow to most fibroids. During MR-HIFU treatments, ultrasound waves are focused in a focal point in the tumour under MR-guidance, resulting in local temperature elevations of at least 57–60 °C. Due to protein denaturation at these elevated temperatures, subsequent tissue necrosis occurs. MR-guidance is used for treatment planning, for temperature measurements in the targeted tissue, and for evaluation of treatment results. The MR-HIFU patient table is integrated with a 1.5 Tesla MRI scanner, forming a closed-loop feedback system. Because of precise targeting guided by MR imaging, no damage is caused to surrounding uterine tissue and other organs. MR-HIFU is currently approved for the treatment of uterine fibroids and under investigation for the treatment of breast cancer, prostate cancer and brain lesions, and pain palliation in bone metastases [7, 8]. During MR-HIFU treatments, proton resonance frequency shift (PRFS) MR thermometry is performed to monitor temperature changes in the targeted and surrounding tissue. However, the temperature monitoring based on PRFS is very sensitive to patient motion and it may also be affected by alterations of breathing patterns [9]. Because the size of each ablation is small (mm in diameter) and cooling times between sonications need to be applied, relatively long treatment durations are required to ablate sufficiently large volumes of tumours cells, making treatments uncomfortable for patients to sustain. Moreover, these treatments may be painful in conscious patients, resulting in discomfort, causing patients to move. Synchronisation of the MR-HIFU treatment is then impossible or the treatment even needs to be aborted. Currently the efficacy and safety of HIFU treatments of uterine fibroids is not widely accepted [10–12]. Female patients are increasingly seeking less invasive treatment options than surgery, motivated by the increased chances of fertility preservation and reduced procedural recovery time. General anaesthesia or epidural anaesthesia has often been used to provide optimal treatment conditions for the radiologist and comfort to the patient [13, 14]. Due the shortage of anaesthesiologists [15], new techniques, such as sedation, have been developed to treat these patients. Sedation is performed to prevent the patient from having MR-HIFU associated deep visceral pain [16] and discomfort caused by “hot” sensations on the skin. Uncontrolled sedation techniques, using a combination of a hypnotic and an opioid, are widely used but are commonly characterised by instable breathing patterns due to variations in pain stimuli, hypnotic dosing, and duration of effects of short acting opioids. An ideal sedation technique for MR-HIFU treatment should result in an appropriate depression of the level of consciousness to enable patients to undergo longer procedures (up to 270 min) in a stable cardiovascular condition, with a regular and stable breathing pattern in order to allow the MR signal to be adequately synchronised with the HIFU signal and with adequate analgesia. Propofol, 2,6-diisopropylphenol [17, 18] is an ultra-short acting hypnotic agent, which does not provide any analgesia. Esketamine is a dissociative anaesthetic providing sedation, analgesia, and amnesia. In anaesthetic doses it may provoke hallucinations as unwanted side effect. The unique catecholamine uptake inhibiting properties cause a sympathomimetic effect resulting in respiratory and cardiovascular stimulation and stability. This is in contrast to other strong analgesic drugs, which are associated with hypoventilation and hypotension. A combination of propofol and esketamine has been documented and described in settings in emergency department sedation and interventional radiology [19–21]. We studied whether a moderate-to-deep sedation technique based on a combination of low-dose propofol and esketamine could provide acceptable and safe conditions for MR-HIFU.

## Methods

### Study population and design

Twenty female patients were included in a pilot study to evaluate the feasibility of moderate-to-deep sedation using propofol and ketamine from August 2013 to November 2014 to provide suitable working conditions for MR-HIFU ablation for uterine fibroids. Approval from the Medical Ethical Committee (protocol number 13-078/C) of the University Medical Center Utrecht was obtained and the patients gave their informed consent. The primary outcome was efficacy; the percentages of patients in whom suitable working conditions allowing adequate synchronisation of the breathing pattern with the MR-HIFU targeting were achieved. Efficacy means the creation of conditions necessary to safely facilitate the completion of a procedure through attenuation of pain, anxiety, and movement with amnesia or decreased awareness [22]. The secondary outcomes were related to patient sedation safety. The incidences of adverse events were recorded as apnea, laryngospasm, hypotension, bradycardia, and airway obstruction. A trained sedation practitioner provided moderate-to-deep sedation as a sole responsibility and was not involved in the procedure itself. The medical condition of all patients was assessed according to the hospital sedation screening protocol. Sedation inclusion criteria used for selection of suitable patients were age ≥18 years, American Society of Anesthesiologists (ASA) I-III, and compliance with fasting policy guidelines prior to procedure. Exclusion criteria: allergy against soy, egg, peanuts, esketamine; the presence of metallic implants; or other incompatibility with MRI (e.g. permanently implanted pacemakers). All procedures were executed in a day case setting, where patients were discharged the same day. The procedure time was defined as the period from the start of procedural sedation and analgesia (PSA) until the patient was awake, with circulatory and ventilatory parameters having returned to normal.

### Preparation and treatment

An 18-gauge (1.3 × 48 mm) intravenous cannula was applied in each patient for continuous infusion of 0.9% saline solution. Patients were pre-medicated with paracetamol 1000 mg, diclofenac 75 mg intravenously and oxycodone 5 mg orally, unless contraindicated. All patients were positioned in the prone position. Patients’ vital signs were continuously monitored (S/5™MRI compatible monitor Datex-Ohmeda Finland) with a three-lead electrocardiogram (ECG), pulse oximetry (SpO_2_), non-invasive blood pressure (NIBP) measurement measured at 5-min intervals or more frequently when needed, and continuous capnography (Smart CapnoLine Oridion Capnography Inc., Needham, MA). All patients received supplemental oxygen (2 L/min) by nasal cannula. All data were electronically recorded (AnStat). Trained sedation practitioners provided PSA, in accordance with a standardised protocol. Low-dose propofol, 5 mg/kg/h (Propofol-Lipuro, Braun, Melsungen, Germany) and esketamine 0.2 mg/kg/h (25 Multi-dose Eurocept BV Ankeveen, the Netherlands) were administered continuously with an infusion pump (Alaris Medical UK Ltd., UK). Just before the start of the MR-HIFU treatment, a single intravenous injection of esketamine 0.25 mg/kg was given. The sedatives were titrated such that a continuous, regular breathing pattern was achieved, in order to allow synchronisation with the MR-HIFU procedure. Demographic details, propofol and esketamine administration, and procedure duration time were recorded. The observer assessment of alertness/sedation (OAA/S) scores were assessed and recorded every 10 min throughout the procedure. OAA/S scores are based on a combination of observations of the resting patient and patient responses to verbal commands with increasing intensity and ranges from 1 (does not respond) to 5 (alert) [23].

### Post-treatment follow-up

After the procedure, all patients were observed in the recovery room for at least 1 h, using a minimum of ECG, NIBP, and SPO_2_ monitoring. The modified Aldrete [24] recovery score was recorded on arrival and every 10 min thereafter. Numeric rating pain scores were recorded and evaluated every 10 min with the use of a visual analogue scale (VAS) 0–10 [25]. Patients were discharged when full consciousness had been regained, when the vital signs (heart rate, pulse oximetry, NIBP) were at the baseline values, when the pain intensity VAS was ≤3, and when the overall recovery (modified Aldrete scale) scores were ≥9 and at least stable for a minimum period of 60 min. Following discharge from the recovery room, patients were transferred to the ward, where they were further clinically observed for the next 3 h and then discharged homewards. Discharge criteria required that the patient was awake and alert with stable vital signs, was able to ambulate without assistance and was free of side effects of the drugs employed during the procedure, and had a VAS ≤3 score. A patient’s clinical outcome follow-up, according to the complication list of the Dutch Society of Anaesthesiology (Additional file 1), was analysed after 24 h, in which the patient completed a questionnaire. The overall satisfaction was rated as never again, insufficient, sufficient, good, or excellent. In addition, pain, unwanted effects (headache, drowsy), complications, the incidence of hallucinations, and the speed of recovery were assessed.

### Complications

PSA-related complications during and after the procedure were defined as severe for hypertension (the diastolic blood pressure >110 mmHg and systole >180 mmHg, for at least 5 min), or hypotension (defined as a mean blood pressure of ≤60 mmHg for at least 5 min), for tachycardia or bradycardia (increase or decrease in heart rate by 30% from baseline, defined as a first measurement before any change occurs), and for oxygen desaturation (SPO_2_ < 92% for at least 5 min). Corrective measures were taken if necessary at the discretion of the sedation practitioners. In addition, upper airway obstruction/laryngospasm (defined as an inspiratory stridor associated with a decrease in arterial oxygen saturation), aspiration (defined as positive when at the slightest clinical suspicion of aspiration a suction catheter was introduced in the trachea and fluid could be aspirated), and nausea (secondary objectives) were also recorded as a severe complication.

### Statistical analysis

Statistical analysis was performed with IBM SPSS statistics, version 23 software (SPSS INC, Chicago, IL). The incidences of the primary outcome, as well as of adverse events are reported as percentages (95% confidence interval (CI)). The correlation between the treatment duration and duration of sleep after the treatment was analysed using linear regression analysis.

## Results

### Patient data

From August 2013 until November 2014, 20 patients received a combined propofol/esketamine sedation according to the protocol, for MR-guided high-intensity focused ultrasound (MR-HIFU) for uterine fibroids procedures. The patients were generally in good health: ASA I: eight patients (40%) and ASA II: 12 patients (60%). Further patient’s demographic data and characteristics are given in Table 1.

**Table 1:** Patient demographic data

	Age	BMI	Duration treatment/Min.	Propofol dose in mg.	Esketamine dose in mg.
Mean	42.4	22.5	240.2	1308.8	42.3
Max	53	29.7	295	1970	87.9
Min	32	17.7	140	692	23.6

Age: in years

*BMI* body mass index: kg/m^2^, *Min* minutes, *mg.* milligram

### Primary outcome and safety

Our sedation procedure resulted in a stable spontaneously, regular and effortless breathing pattern and in a relaxed, non-moving patient, allowing excellent synchronisation of the breathing pattern with the MR-HIFU procedure in all patients (100%). The sedation technique with low-dose propofol and esketamine that was selected for this MR-HIFU treatment resulted in stable hemodynamic and ventilatory conditions, and no pain and discomfort to the patient during this procedure. A stable breathing pattern proved to be essential to allow an optimal synchronisation between the continuous MR signal and the high-intensity ultrasound targeting, which is vital for an optimal treatment. All therapeutic procedures could be finalised without interruption due to faulty synchronisation of the MR signal and the HIFU targeting. Sedation-related adverse events were not reported during and after our treatments. The effects of the sedation technique based on mean NIBP, heart rate, SpO_2_, capnography, respiration rate, and OAA/S score are depicted in Table 2. A regular breathing pattern was obtained 5 min after the esketamine was administered. There was no morbidity or mortality during and following the MR-HIFU procedures. All patients recovered well and were discharged the same day. All MR-HIFU treatment procedures could be carried out efficiently and according to plan. No hallucinations were observed and reported during and after the treatment. In our study the patient was relaxed, was not moving, and had no pain, thereby allowing excellent synchronisation with a stable spontaneously regular and effortless breathing pattern during the MR-HIFU procedure.

**Table 2:** Sedation-related events during MR-HIFU treatment (*N* = 20)

	Heart rate Min max mean	SpO_2_ Min max mean	Capnography Min max mean	Resp. rate Min max mean	Mean NIBP Min max mean	OAA/S score Min max mean
1 min	50 112 79.9	94 100 98	4.5 6.0 5.2	10 20 14	79 112 98	5 5 5.0
30 min	54 102 73.2	96 100 98	4.6 5.8 5.4	9 18 14	71 110 86	3 4 3.8
60 min	56 100 72.4	95 99 97	4.5 6.2 5.5	9 20 14	62 105 79	2 5 3.1
90 min	50 90 71.0	94 99 97	4.5 6.2 5.7	8 20 13	62 90 74	2 4 2.6
120 min	54 92 71.7	94 99 97	4.9 6.3 5.8	8 24 15	64 94 79	2 4 2.5
150 min	50 96 71.7	94 100 97	5.0 6.2 5.7	8 24 15	62 102 81	2 3 2.5
180 min	48 92 71.4	94 100 97	4.4 6.1 5.6	8 25 15	68 95 80	2 3 2.6
210 min	50 90 72.0	95 100 97	4.9 6.1 5.5	8 24 14	68 94 84	2 4 2.8
240 min	54 90 72.0	96 100 98	5.0 5.9 5.5	9 24 15	67 99 85	2 4 3.1
270 min	54 88 77.2	96 100 98	5.3 5.9 5.5	12 18 14	80 101 87	3 5 4.0

*Min* minutes after start patient records registration, *HR* heart rate/minute, *SpO*_*2*_ saturation of peripheral oxygen in %, *Capnography* capnography in vol %, *Resp. rate* respiratory rate/minute, *Mean NIBP* mean non-invasive blood pressure in mmHG, *OAA/S* observer’s assessment of alertness/sedation

### Recovery

All patients achieved the maximum Aldrete score of 10 points within the first 60 min, and the intensity of pain (VAS score) after the procedure differed from patient to patient (Table 3). Eighteen out of 20 patients indicated a VAS score of <3 within 30 min. After 60 min, one patient had still a VAS 8 score (due to back pain) and a second one a VAS 9 (due to skin burns). Both were successfully treated with intra venous morphine. After 240 min all patients could be discharged from the hospital with a VAS score ≤3.

**Table 3:** Patient VAS and Aldrete score

		VAS score	Aldrete score
	*N*	Min max mean	Min max mean
After 10 min	20	0 8 0.7	6 10 8.35
After 30 min	20	0 8 1.15	8 10 9.65
After 60 min	20	0 7 1.15	9 10 9.8
After 240 min	20	0 3 0.6	9 10 9.85

*VAS* visual analogue scale score, *N* number of patients, *Min* minimum, *Max* maximum, *x min x* minutes after the sedation

### Post-treatment evaluation

Fourteen patients rated the overall sedation satisfaction for the MR-HIFU treatment as excellent, and six patients rated the overall satisfaction as good. Fourteen patients could resume their everyday activities within 2–4 h, three patients within 6 h, two patients in less than 12 h, and one patient resumed her daily activities after 72 h following discharge from the hospital (Table 4). Three patients indicated to have had a headache or migraine-like headaches. Three patients were tired following discharge and drowsy, and one patient noted difficulty in passing stools for 24 h. In linear regression analysis, a longer duration of the treatment in minutes was associated with a shorter recovery time after the treatment (*R* = 0.810, *p* < 0.001).

**Table 4:** Patient resumation of daily activities

Resumation daily activities after *x* minutes	Patients (*N*)	Percent (%)	Cumulative percent (%)
0 min	*N* = 2	10	10
100 min	*N* = 1	5	15
120 min	*N* = 7	35	50
150 min	*N* = 1	5	55
240 min	*N* = 4	20	75
270 min	*N* = 1	5	80
300 min	*N* = 1	5	85
360 min	*N* = 2	10	95
720 min	*N* = 1	5	100

## Discussion

MR-HIFU treatment of uterine fibroids is a promising technique for treatment of uterine fibroids. The main advantages, when compared to the conventional surgical approach are that it is a non-invasive treatment allowing quick recovery in day care, offering the advantage of an increased likelihood to preserve fertility for a group of young women. For an MR-HIFU treatment, which may take up to 4 h or longer, to be successful, excellent working conditions are required. This means a patient lying still during the whole procedure and a strictly regular breathing pattern allowing optimal synchronisation between the continuous MR signal and the high-intensity focused ultrasound-targeting procedure. Previous attempts to apply moderate-to-deep sedation using hypnotics and/or opioids for this long lasting procedure, which is uncomfortable and often painful to undergo, have failed. The main reason is a global shortage of trained medical sedation practitioners and professionals, including anesthesiologist to administer procedural sedation and analgesia [26–28].

The preferred method to allow this MR-HIFU procedure is therefore general anaesthesia including muscular relaxation and artificial ventilation in most cases [29]. The presence of the pain, movements, and no regular breathing may complicate the treatment procedure. The patient may not be able to lie still for a prolonged period of time or treatment-induced involuntary motion may occur if the treatment is performed, while the patient is conscious. Patient motion may hamper the MR images that are used for treatment monitoring. In the present study, we report the clinical observational study of the use of moderate-to-deep sedation to allow MR-HIFU therapy directed at ablating large volumes of uterine fibroids. Using a combined technique with propofol and esketamine infusion, we showed that moderate-to-deep sedation with this particular technique resulted in regular breathing patterns and adequate conditions to allow an undisrupted MR-HIFU procedure for up to 270 min. The stable breathing pattern allowed for an optimal synchronisation between the continuous MRI signal and the high-intensity focused ultrasound targeting in all patients, which is vital for an optimal treatment. The technique also caused comfort to the patient with a limited recovery time and good patient satisfaction. Our study showed that ablation of uterine fibroids using MR-HIFU with propofol/esketamine sedation was safe and feasible without sedation-related adverse effects or complications that occurred during and/or after the treatment. In other studies of sedation for HIFU treatment (with midazolam and fentanyl) [30–32], the incidences of mild and moderate pain in the abdomen were comparable to our findings after the procedure. The level of safety achieved in the present study may be the result of strict adherence to a sedation protocol, careful patient selection and monitoring, experience in the use of drugs, and extensive staff training. Mortero et al. [33] confirms this in his study, where he compared the effects of propofol and small-dose ketamine to solely propofol, that a mixture of propofol and ketamine provides an adequate and safe sedation and ventilation level during monitored anaesthesia care, in elective ambulatory surgery. Frizelle et al. [34] showed in his study that the side effects of propofol during spinal anaesthesia could cause dose-related cardiovascular and respiratory depression. The addition of a small-dose ketamine infusion to propofol increased hemodynamic stability, and less respiratory depression, similar to our current finding. Frey et al. [35] reviewed propofol esketamine published studies and concluded that the combination of ketamine and propofol in bolus form provides safer and more efficacious sedation, with sufficient stability of vital signs. This is in accordance with our findings. Twenty-four patients, who prefer treatment without anaesthesia or sedation, by using low-dose sonographically guided HIFU treatment, were alternatively treated. Nevertheless, consequently mild pain, heat sensation, and discomfort occurred during and after the treatment in the study of Cho and Leung [36]. Our results show that we had fewer longer-term side effects than Fukunishi et al. [37] reported. In his study, five (out of 20) patients took longer than 3 days to return to their normal daily activity and three patients experienced discomfort for more than 1 week due to mild buttock pain.

A possible disadvantage of performing HIFU treatments under deep sedation is the patient’s disability to communicate in case of pain or discomfort. This could lead to a higher adverse event rate or even major complications. In our study, redness of the skin was observed in several patients after concluding the treatment and is due to accumulative heating in the subcutaneous adipose tissue in spite of long cooling times (2–10 min) in between consecutive sonications. To resolve this issue and to shorten cooling times, which in turn increases the efficiency of the treatment and thereby the total treatment volume, a skin cooling system was designed by Philips. This device was integrated into the Sonalleve V2 HIFU-table and was tested previously by our group with very promising results [38]. Careful treatment planning using anatomical MR images and real-time MR thermometry prevented major adverse events, such as sacral nerve damage.

This study had several limitations. The number of recorded cases was relatively small, limited due to the randomised study, and the population was treated in only a single centre. Although the results of this study may help to understand and manage similar clinical situations, any attempt of other scenarios should be performed very carefully and confirmed in clinical studies. This sedation technique in combination with a HIFU treatment may create new and innovative ways to treat patients with liver and kidney tumours. Finally larger studies are necessary, for a long-term evaluation of sedation-related outcomes, efficacy, and side effects of esketamine and propofol sedation.

## Conclusions

The use of a combination of propofol and esketamine for procedural sedation and analgesia reduces the discomfort and pain during MR-HIFU treatments of uterine fibroids to an acceptable level according to the opinion of the patients. This technique also caused cardiovascular and respiratory stability and allowed easy synchronisation of the breathing pattern with the MR-HIFU signal to provide optimal treatment conditions for the interventional radiologist. Based on our preliminary results, we consider our esketamine and propofol sedation technique for MR-HIFU treatment of uterine fibroids to allow optimal treatment conditions, without major adverse events, to be safe and efficient with a short recovery time for MR-HIFU treatments of fibroid tumours. The technique may be considered as an acceptable alternative to general anaesthesia with muscular relaxation and securing the airway with a laryngeal mask or endotracheal tube.

## Additional file

Additional file 1: Patient satisfaction from after 24 h. (DOCX 109 kb)

## Abbreviations

MR-HIFU: Magnetic resonance high-intensity focused ultrasound
PSA: Procedural sedation and analgesia
