# Supplementary material for: Moderate-to-deep sedation technique, using propofol and ketamine, allowing synchronised breathing for magnetic resonance high-intensity focused ultrasound (MR-HIFU) treatment for uterine fibroids: a pilot study
Source: J Ther Ultrasound. 2017 Feb 9;5:8. doi: 10.1186/s40349-017-0088-9 (PMC5299783; doi:10.1186/s40349-017-0088-9)
Supplement: Additional file 1: — Patient satisfaction from after 24 h. (DOCX 109 kb) [file 40349_2017_88_MOESM1_ESM.docx]

Additional file 1: Patient Satisfaction form after 24 hours

**
